# Pharmacological Targeting of Cell Cycle, Apoptotic and Cell Adhesion Signaling Pathways Implicated in Chemoresistance of Cancer Cells

**DOI:** 10.3390/ijms19061690

**Published:** 2018-06-06

**Authors:** Dauren Alimbetov, Sholpan Askarova, Bauyrzhan Umbayev, Terence Davis, David Kipling

**Affiliations:** 1 Laboratory of bioengineering and regenerative medicine, Center for Life Sciences, National Laboratory Astana, Nazarbayev University, 53 Kabanbay Batyr Ave, Z05H0P9 Astana, Kazakhstan; shaskarova@nu.edu.kz (S.A.); bauyrzhan.umbayev@nu.edu.kz (B.U.); 2 Division of Cancer and Genetics, Cardiff University School of Medicine, Heath Park, Cardiff CF14 4XN, UK; davist2@Cardiff.ac.uk (T.D.); kiplingd@cardiff.ac.uk (D.K.)

**Keywords:** chemoresistance, cell cycle, apoptosis, cell adhesion, small molecules

## Abstract

Chemotherapeutic drugs target a physiological differentiating feature of cancer cells as they tend to actively proliferate more than normal cells. They have well-known side-effects resulting from the death of highly proliferative normal cells in the gut and immune system. Cancer treatment has changed dramatically over the years owing to rapid advances in oncology research. Developments in cancer therapies, namely surgery, radiotherapy, cytotoxic chemotherapy and selective treatment methods due to better understanding of tumor characteristics, have significantly increased cancer survival. However, many chemotherapeutic regimes still fail, with 90% of the drug failures in metastatic cancer treatment due to chemoresistance, as cancer cells eventually develop resistance to chemotherapeutic drugs. Chemoresistance is caused through genetic mutations in various proteins involved in cellular mechanisms such as cell cycle, apoptosis and cell adhesion, and targeting those mechanisms could improve outcomes of cancer therapy. Recent developments in cancer treatment are focused on combination therapy, whereby cells are sensitized to chemotherapeutic agents using inhibitors of target pathways inducing chemoresistance thus, hopefully, overcoming the problems of drug resistance. In this review, we discuss the role of cell cycle, apoptosis and cell adhesion in cancer chemoresistance mechanisms, possible drugs to target these pathways and, thus, novel therapeutic approaches for cancer treatment.

## 1. Introduction

Chemotherapy remains the major treatment in cancer therapy, although the molecular mechanisms causing sensitivity or resistance to chemotherapeutic drugs in different tumor types are still unclear. Thus, determining the optimal chemotherapeutic regime for a given cancer type is problematic, and can result in very different outcomes for the individual. For instance, testicular cancers respond well to chemotherapy resulting in around 80% positive outcomes [1], while others, such as non-small cell lung cancer (NSCLC), have only a 30% response rate to cytotoxic platinum based chemotherapy leaving the remaining 70% of patients with little to no benefit or, indeed, side effects related to drug toxicity [2]. Most chemotherapeutic agents cause DNA damage and activate a complex signaling network resulting in cell cycle arrest and/or apoptosis. During the course of tumorigenesis and tumor progression, nearly all cancer cells disrupt components of the DNA damage response (DDR) pathway [3]. The disruption to the DDR eventually leads to genomic instability that may underlie some aspects of cancer chemoresistance. Thus, the effectiveness of chemotherapy may depend upon DDR differences between normal and tumor cells [4,5].

When DNA damaging agents are used to treat cancer, some clones within the cancer tissue up-regulate the expression of specific genes that either activate, or suppress, signaling networks regulating cell cycle arrest or DNA repair. These alterations may result in resistance of cells to drug-induced death signals. Consequently, this mechanism acts as a positive selection pressure in favor of recurrent tumors originating from drug resistant clones [6]. Thus, studying specific resistance mechanisms and developing new therapeutic strategies directed against these are extremely important in improving the effectiveness of chemotherapy and, hence, patient survival. In this review we focus on three main mechanisms involved in chemoresistance, alterations of which we believe will play an important role in improving the effectiveness of cancer therapy.

### 1.1. Cell Cycle

Regulation of the cell cycle by targeting cell-signaling pathways is a relatively new area for cancer treatment, and targeting cell cycle phases and checkpoints could provide unique opportunities and promise for the improvement of cancer treatment. Cell cycle progression has five known phases: G0 (gap 0), G1 (gap 1), S (DNA synthesis), G2 (gap 2), and M (mitosis). In between these phases are checkpoints at which times the integrity of cellular components and the fidelity of DNA synthesis are monitored. Two important checkpoints are at the G1/S and G2/M boundaries [7].

Tumor cells tend to accumulate alterations in components of the cell cycle machinery which results in an impaired ability to respond to DNA damage, in particular by halting cell cycle progression, and an abnormal distribution of cells during cell cycle progression is a hallmark of human cancer. As many anti-cancer drugs are DNA damaging agents, a reduced ability of the cell cycle machinery to respond to DNA damage may result in chemoresistance. To date, there are a number of cancer therapeutic strategies focused on cell cycle regulation. However, many of the proposed drugs failed to selectively kill cancer cells without also having major cytotoxic effects on normal cells; in other words, they had a small therapeutic window [7,8,9]. Therefore, a new generation of drugs targeting cell cycle features that are more specific to cancer cells is needed.

### 1.2. Apoptosis

Apoptosis is a mechanism of programmed cell death whereby cells with extensive DNA damage can be eliminated [10]. It was in the early 1970s when Kerr et al. first suggested that apoptosis could be vital for eliminating malignant cells, hyperplasia and tumor progression [10]. Therefore, triggering apoptosis in cancerous cells may play an important role in cancer therapy, and malignant tumor cells need to be selectively killed so healthy cells remain untouched for further proliferation [11]. However, another hallmark of cancer is the ability of tumor cells to avoid apoptosis during chemotherapy. There are three main known mechanisms by which cancer cells acquire apoptosis resistance: (1) a disruption in the balance between pro- and anti-apoptotic proteins, (2) an impairment of signaling through death receptors and (3) a reduction in the function of caspases [12]. Many strategies are being developed to target these pathways in order to counter chemoresistance mechanisms in cancer, and this is a fruitful field for further investigations.

### 1.3. Cell Adhesion

Cell adhesion is another mechanism essential for cell growth, cell migration and cell differentiation [13,14]. Cell adhesion is an interaction between individual cells, or between cells and extracellular matrix components, and important molecules involved in these interactions are the cellular adhesion molecules (CAMs). CAMs are implicated in processes as varied as cellular recognition and communication, signal transduction, embryogenesis, immunity, apoptosis and inflammation [15]. CAMs are thought to be involved in the development of metastasis as they contribute to the spread of metastatic tumor cells into the blood or lymphatic circulation due to the loss of intercellular adhesion, and there is strong evidence suggesting that CAMs could be associated with invasion in many human cancers [16,17]. The role of intercellular interaction in cancer cell survival during DNA damage due to radio or chemotherapy was first described by Durand and Sutherland [18]. These diverse functions identify CAMs as an attractive target for cancer therapy.

## 2. Cell Cycle as a Target for Overcoming Chemoresistance during Cancer Therapy

### 2.1. Cyclins and Cyclin Dependent Kinases

Cell cycle progression is regulated by cyclins through activating cyclin-dependent kinase (CDKs). CDKs are upregulated by cyclins (A, B, D and E) and downregulated by cyclin-dependent kinase inhibitors such as p16^INK4a^ and p21^Cip1^ [19]. To date, there are nine known structurally related CDKs (CDK1–CDK9) although their role in cell cycle has not been fully explored. Furthermore, CDKs play an important role in apoptosis and are up or downregulated in many cancers including lymphoma, lung cancer, leukemia, pancreatic tumors and hepatocellular carcinoma [20]. Several cyclins have been identified that control cell cycle progression via activation of CDKs. D-cyclins are activated in G1 phase to direct phosphorylation of the cell cycle inhibitor pRb which in turn inactivates regulatory functions of cells allowing their progression into S phase. Cyclin E accumulates at the G1/S phase and activates CDK2, promoting progression through the G1 interval. Cyclin A promotes cell cycle progression through G2—accumulates during S and goes down during G2. Further, cyclin B and CDK1 interact to drive cells into mitosis [20,21].

Given the importance of the cyclins and CDKs for cell cycle control, these make attractive targets for chemotherapeutic intervention with pharmacological inhibitors (herein called CKIs) proposed for cancer treatment [22]. To date there are 11 known classes of CDK ATP competitive inhibitors, such as staurosporine, flavonoid, purine indole, pyrine, pyrimidine, indirubin, pyrazole, thiazole, paullone and hymenialdisine derivatives [23]. These CKIs target various cyclin/CDK complexes showing some positive results in vitro and in vivo. For example, the CKI, roscovitine, sensitized glioblastoma cells to tumor necrosis factor-related apoptosis-inducing ligand (TRAIL)-mediated apoptosis in vitro by targeting CDK2/cyclin B, E or A complexes [24] and milciclib inhibited cell proliferation, downregulated CDK4/Rb transduction pathway markers, and induced cell death by autophagy through inhibition of CDK2/cyclin A, CDK7/cyclin H and CDK4/cyclin D1 complexes in glioblastoma cells [25].

First generation CKIs used in chemotherapy (e.g., flavopiridol) resulted in G1 or G2 phase cell arrest in vitro in hematological cancers as a result of CDK7 and CDK9 inhibition and showed good activity pre-clinically, although they possessed off-target toxicities in vivo, in particular neutropenia [26]. A positive effect for flavopirdol to enhance cytotoxicity in glioblastoma cells was observed when used in combination with temozolomide [27]. Resistance to temozolomide occurred due to activation of G2 checkpoint mediated DNA repair, although it was reported that CKIs prevented temozolomide resistance by suppressing the DNA repair mechanism at the G2/M phase of the cell cycle [27].

Further, the small molecule CGP 75414A induced cell cycle arrest and apoptosis in human leukemic cell lines, and caused a modest G2/M arrest, apoptosis via Poly(ADP-ribose Polymerase (PARP) cleavage and mitochondrial damage in U937 monocytic human cancer cells by inhibiting the activity of CDK2 and CDK4 [22]. In addition, novel small molecule derivatives (BA-12 and BP-14) of the roscovitine were shown to induce accumulation of hepatocellular carcinoma cells in G2/M and S/G2 phases of the cell cycle, suggesting that both BA-12 and BP-14 possess antiproliferative activity [28]. These molecules did not cause resistance in several hepatoma cell lines and no enhanced viability of hepatocellular carcinoma cells was observed after long-term treatment [28].

It is important to note the role of nuclear factor kappaB (NF-κB) in chemoresistance mechanisms involving Cyclin/CDK complexes [29]. NF-κB may induce chemoresistance by regulating the cell cycle and exerting anti-apoptotic properties. Some data has shown that inhibition of NF-κB activity suppresses cell cycle progression, cell anti-apoptosis (anti-apoptotic proteins Bcl-2, X-linked inhibitor of apoptosis (XIAP), and B-cell lymphoma extra-large (Bcl-XL) and chemoresistance by reducing the concentrations of cyclins A, B, and D1, and CDK4 and CDK6, the major proteins associated with cell cycle [29].

Overall, cyclins and CDKs represent promising targets that may potentially improve the efficacy of standard chemotherapeutic cancer agents when the latter are used in combination with novel CKIs.

### 2.2. The DNA Damage Responsive p53 Pathway

*Tp53* is a tumor suppressor gene encoding p53 that regulates cellular proliferation and apoptosis by activating several molecular pathways [30]. Recent findings suggest that the p53 signaling pathway is involved in chemosensitization of cancer cells to DNA-damaging agents through DNA damage response sensors ataxia telangiectasia mutated protein (ATM) and ataxia telangiectasia and Rad3-related protein (ATR) and their downstream cell cycle regulator checkpoint kinases 1 and 2 (Chk1 and Chk2) [31,32,33]. Chk1 and Chk2 kinases differ in structure although they exert similar functions in mediating cell cycle in response to genotoxic stress. Cell cycle arrest upon DNA damage is regulated by the p53-p21-dependent G1 checkpoint [31] and the Chk1-Cdc25-dependent G2 checkpoint [32,33].

The role of p53 in cancer has been extensively studied [34,35,36]. The importance of p53 upstream activation mechanisms and the kinases ATM and ATR in regulating DNA damage in response to double-strand breaks is also well known [37]. However, the specific alterations in these genes that contribute to drug resistance during chemotherapy still remain obscure. p53 is an important tumor suppressive factor, mutation of which plays an important role in many drug resistant mechanisms. For example, p53 activates the ATP-binding cassette transporter MDR1 (multidrug resistance 1) to cause resistance. *Tp53* mutations are also associated with elevated levels of MDR-associated protein 2 (MRP2) and breast cancer resistance protein (BCRP), as well as high glutathione levels. Glutathione conjugates cisplatin as a substrate of ABC transporters, leading to cisplatin efflux and resistance [34,38]. High levels of NF-κB2, Fos proto-oncogene protein (FOS) and MYC proto-oncogene protein (MYC) and the transactivation of nuclear transcription factor Y (NF-Y) in tumors that have mutated *Tp53*, also result in chemoresistance by activating the expression of target genes involved in cell survival, signal activation, and apoptosis resistance [34].

A combination therapy therefore using two or more chemotherapeutic drugs or inhibitors to increase the sensitivity of cancer cells to chemotherapeutic drugs could be used to treat *Tp53*-mutated cancers. Such combination therapy is predominantly focused on p53-deficient cancer cells as *Tp53* mutation causes G1 checkpoint impairment [35] therefore leaving cancer cells to rely on G2 checkpoint for DNA repair and survival. This opens new possibilities for using G2 checkpoint inhibitors as chemosensitizers for p53-deficient cancer cells [36,39] with several checkpoint kinase inhibitors being currently tested in clinical trials [40]. The clinical usage of one of the main checkpoint inhibitors, UCN-01, was limited due to its destruction by plasma proteins in vivo, therefore new small molecule inhibitors of Chk1 or Chk2 are needed that avoid this issue so as to test their therapeutic potential for sensitizing p53-deficient cancer cells [41]. The G2 checkpoint inhibitor CBP-93872 significantly blocks the activity of ATR and Chk1 phosphorylation induced by chemotherapeutic drugs oxaliplatin or cisplatin [42]. The effect of CBP-93872 was seen as suppression of the G2 checkpoint by inhibiting DSB-dependent ATR activation [43,44], possibly improving the effect of DNA damaging agents in p53-deficient cancer cells. This molecule may have a non-toxic effect on healthy cells with activated p53 and p21 pathways that may indicate CBP-93872 as an effective chemosensitizer when used in combination with chemotherapeutic drugs such as oxaliplatin, cisplatin, gemcitabine, or 5-FU [42].

The tyrosine kinase WEE1 is highly expressed in many cancer types and plays a role in cell cycle progression via the G2 checkpoint [45]. It is implicated in cancer cell survival in mutated *Tp53* cells and its loss sensitizes such cells to chemotherapy with DNA damaging agents by increasing apoptosis. Chemosensitivity to agents such as cisplatin, gemcitabine and carboplatin was increased when used in combination with the WEE1 inhibitor MK-1775 (otherwise called AZD1775) with tumor growth being reduced in many cancer types; importantly, no additional toxicity beyond that seen with the DNA damaging agents alone was seen [46]. Currently clinical trials using MK-1775 in combination with paclitaxel (NCT02448329) and carboplatin-paclitaxel (NCT02513563) are in progress at this time for cancers as diverse as advanced gastric adenocarcinoma and metastatic solid tumors. MK-1775 is also in trials with taxol for ovarian cancer (NCT02272790, NCT02272790, NCT01357161) [47]. Thus, the above data suggest that targeting of molecular components of the G2 checkpoint may have therapeutic promise in G1 checkpoint defective *Tp53*-mutated cancers.

### 2.3. Targetting Mutated p53

As mutated p53 status is a feature of many cancers, it makes an attractive target for therapy [45,48]. Some mutated p53 forms are very stable and heterodimerize with wild-type p53 and can act in a dominant negative fashion disrupting, or abrogating, most or all normal p53 functions, such as apoptosis or cell cycle arrest [45,49,50]. For example, p53 is degraded by the E3 ubiquitin ligase mouse double minute 2 homolog (MDM2) that is a target of p53 transactivation; however many mutated p53 isoforms do not induce MDM2 resulting in mutated p53 stability [51]. This suggests that directly targeting mutated p53 may be therapeutically effective in many cancers, and some animal models have revealed promising outcomes in tumor regression when wild-type p53 was reactivated in p53 mutated cancers [52]. Although difficult to achieve, the main treatment strategies to date focus on the destabilization or inactivation of mutated p53, or the reactivation of wild-type functions in the mutated p53 protein, such as cell cycle arrest or apoptosis [53]. In this regard, the anti-cholesterol statins deplete misfolded p53 [54], although this was found to be ineffective as a therapy when used to treat prostate cancer [55]. However, pre-clinical studies do show that reactivation of p53 can slow tumor progression making this type of approach worthy of further study [56].

The small molecule PRIMA-1 and its analog PRIMA-1MET (APR-246) restored mutant p53 to a wild-type conformation leading to expression of p53 targets involved in apoptosis, notably Bax, Noxa and Puma [57,58]. In vivo, APR-246 shows impressive apoptotic and cytotoxic effects in p53 mutated SCLC, breast cancer and multiple myeloma, with the drug well tolerated during Phase I/IIa clinical trials [59,60,61]. APR-246 is currently in combination therapy trials with cisplatin, carboplatin or azacytidine for oesophageal, ovarian and myeloid cancers, and results are awaited [45]. In addition, APR-246 when used in combination with the proteasome inhibitor carfilzomibin overcame the chemoresistance to carfilzomibin in triple negative breast cancer [62]. As mutated p53 affects protein homeostasis via the proteasome machinery that can inhibit tumor suppression, these results create opportunities to develop combination therapy using drugs to target mutant p53 with anti-proteasome inhibitors.

Missense mutations in p53 often result in gain-of-function p53 isoforms, thus leading to great interest in the discovery of small molecules that destabilize mutated p53, allowing wild-type p53 to regain its functionality. In this regard the active chemical component in peppers, capsaicin (that has known anti-tumor properties), resulted in the degradation of mutated p53 by activating autophagy and lead to cell death in NSCLC cells [63]. Many mutated p53 forms can stimulate mechanistic target of rapamycin (mTOR) and block autophagic processes that could otherwise be tumor suppressive, leading to anti-apoptotic and pro-proliferative responses in breast and pancreatic cancer [64]. This mTOR stimulation sensitized cancer cells to mTOR inhibitors such as everolimus. Another vegetable compound, phenethyl isothiocyanate, restored wild-type p53 and inhibited tumor growth in a xenograft model [65]. Although not yet used in clinical trials, such naturally occurring p53 restoring dietary compounds may be a valuable addition to the chemotherapeutic arsenal as their in vivo toxicity to normal tissue may be low.

An alternative mechanism to deplete cells of mutated p53 protein complexes is the use of small peptides that prevent the ability of mutated p53 to bind to target proteins, and such peptides enhanced the therapeutic effects of adriamycin and cisplatin by inducing apoptosis [66]. Destabilization of mutated p53 complexes could also be achieved in cancer cells using small molecules PK-083 and PK-7088, resulting in activation of pro-apoptotic Noxa expression and apoptosis [67].

The paragraphs above only give a taste of the possibilities of anti-mutated-p53 therapeutics for chemosensitization; however, these strategies are still at an early developmental stage, although they do show promise for the future.

### 2.4. Aurora Kinase Signaling

The next pathway of interest is Aurora-A kinase (AURKA), a member of the mitotic serine/threonine kinase family involved in mitosis and meiosis during cell proliferation. The known Aurora kinases (A, B and C) share similar amino acid sequences, but their subcellular localization and functions differ. All three Aurora kinases are involved in cell division; however, Aurora-A regulates cell cycle progression by regulating the spindle and mitotic checkpoints. Its main functions are mitotic regulation, promotion of mitotic entry, and cell growth arrest [68]. Overexpression of Aurora-A is linked to breast, ovary, and colon tumors and is shown to act as an oncogene in many in vitro models [69]. Moreover, overexpression of Aurora-A has been associated with radio- and chemoresistance in laryngeal cancer cells [70], cervical cancer [71] and breast cancer [72]. Aurora-A has been suggested to induce chemoresistance in several cancers by reducing apoptosis via activation of the NF-κB/miR-21/PTEN (phosphatase and tensin homolog) signaling pathway [73] and Akt through inhibition of the p53/PTEN cascade [74]. In addition, Sun et al. [75] have demonstrated that Aurora-A can induce radio- and chemoresistance through ATM/Chk2-mediated dysregulation of DNA damage repair networks including pp53, γH2AX, and RAD51. These findings suggest Aurora-A kinase as a possible drug target to improve the outcomes of cancer therapy in many cancer types.

Aurora-A kinase inhibitors currently used in preclinical and clinical studies include MLN8054, PF-03814735, AS703569, MK-0457, MK-5108, MSC1992371A and MLN8237 [68]. Among these small molecule inhibitors MLN8237 was effective in treating acute myelogenous leukemia and chronic myelogenous leukemia in Phase II trials when used in combination with cytarabine [76] and nilotinib [77]. Another Aurora-A kinase inhibitor, MK-5108 (Phase I), inhibits cell growth and induces G2/M arrest in chemoresistant epithelial ovarian cancer stem cells by affecting the NF-κB pathway [78]. Aurora kinase is a key player in mitosis and cancer and has attracted much attention as a therapeutic target for the treatment of leukemia and many other solid tumors [68]. More studies are needed to focus on the further usage of Aurora kinase inhibitors combined with conventional therapies to establish the most effective inhibition dosages.

### 2.5. BRCA1/2

The tumor suppressor genes *BRCA1/2* are frequently mutated in familial breast and ovarian cancer, and around 10% of women diagnosed with these pathologies carry *BRCA1/2* mutations [79]. Furthermore, carriers of *BRCA1/2* mutations were reported to be at increased risk of developing pancreatic and prostate cancers [80]. The BRCA1/2 proteins are involved in several cellular mechanisms such as cell cycle checkpoint control, chromosome remodeling, transcriptional regulation, DNA repair, and apoptosis [79,81]. Additionally, BRCA1/2 are essential for both S and G2/M checkpoints in response to DNA damage caused by either radio or chemotherapy, and play important roles in multiple DNA repair pathways such as homologous recombination (HR) and transcription-coupled nucleotide excision repair (TCNER) [79,81,82].

Therefore, *BRCA1/2*-null cancers are more sensitive to platinum-based DNA damaging agents and to PARP inhibitors [83,84]. Nevertheless, these *BRCA1/2*-null cancers develop resistance over time due to restored BRCA1/2 functions, as secondary mutations of *BRCA1/2* occur in *BRCA1/2*-mutated tumors [85]. An inability to repair DNA makes cells sensitive to DNA damaging drugs, and restoration of DNA repair functions results in acquired resistance to those drugs [86]. It has been suggested that mutations in p53 upregulate *BRCA1* and induce resistance to cisplatin in breast cancer. Further, BRCA1 can activate the transcriptional target TDP2 that pairs with ETS2 and mediates etoposide resistance in mutp53-bearing cells [34]. Studies carried out by Wiltshire et al. [87] revealed that BRCA1 contributes to irofulven (6-hydroxymethylacylfulvene) chemoresistance, an anticancer agent derived from mushroom produced illudin toxins [87].

These established characteristics of BRCA1/2 proteins in chemoresistance indicate that new drugs are needed for BRCA1/2 inhibition, as known effective small molecules only affect BRCA1 indirectly. For example, inhibition of the homologous recombination (HR) pathway proteins RAD52/51 with small molecule D-I03 can specifically inhibit the biochemical activities of RAD52 and suppress growth of *BRCA1* and *BRCA2* null cells [88]. Likewise, PARP inhibitors (e.g., talazoparib, niraparib, olaparib, and veliparib) are also capable of sensitizing tumor cells with impaired HR activity by genomic instability and cell death. Since *BRCA1* and *BRCA2* mutated cells lack HR pathways, such inhibitors improve the effectiveness of chemotherapy in breast and ovarian cancer treatment [89].

### 2.6. Wingless (WNT) Signaling

WNT family proteins are essential for regulating diverse cellular mechanisms including cell proliferation, survival, migration and polarity, as well as cell fate specification and stem cell self-renewal [90]. Two main categories of WNT pathways have been identified to date: canonical WNT signaling (1) dependent on the transcriptional activity of β-catenin and non-canonical WNT signaling (2) which lacks β-catenin transcriptional activity [91]. WNT5A is a non-canonical member of the WNT family and is a tumor autocrine/paracrine factor highly expressed in many cancer types [91]. Upregulation of WNT5A is associated with breast cancer [92], prostate cancer [93], melanoma [94] and pancreatic cancer [90], indicating its oncogenic role in these cancers.

However, WNT5A is thought to induce chemoresistance in pancreatic cancer through enhanced PI3K/Akt signaling that affects the G1/S phase transition [90]. Furthermore, WNT5A was highly expressed in BRAF inhibitor (BRAFi)-resistant melanoma tumors [95]. The drug resistance mechanism appears to be that high levels of WNT5A activates signaling through Fzd7 and Ryk receptors that induce PI3K/Akt signaling resulting in increased growth and therapeutic resistance to BRAF inhibitors [95]. Further, WNT5A activates the WNT/protein kinase C (PKC) signaling pathway that is highly expressed in many cancers and causes chemoresistance by partly activating WNT/β-catenin signaling [96]. This was confirmed by in vitro studies where the PKC inhibitor GF109203X significantly inhibited WNT5A induced cell migration, invasion, and clonogenicity in A549 and A549/DDP (diamminedichloroplatinum) lung cancer cells, indicating a clear WNT5A role in promoting lung cancer cell mobility through WNT/PKC noncanonical pathway activation [97].

A recent study using WNT5A knockdown showed an increase of cells in G0/G1 phase and a decreased cell number in S phase, which enhanced the chemosensitivity of pancreatic cancer cells to gemcitabine [90]. Another study reported that WNT5A contributed to drug-resistance by enhancing anti-apoptosis ability in pancreatic cancer cells [98]. Since gemcitabine is a cell cycle specific drug, these studies have found that WNT5A mediated gemcitabine chemoresistance was via the regulation of cell cycle, which suggests WNT5A as an effective gemcitabine chemoresistance predictor and a target for chemotherapeutic response in pancreatic cancer.

### 2.7. The p38 MAP Kinase Pathway

The p38 mitogen activated protein kinases (herein called p38) are a family of serine/threonine kinases classified as “stress-activated” kinases in response to a variety of extracellular stimuli in different organisms. Immediate downstream of p38 is mitogen-activated protein kinase activated protein kinase 2 (MAPKAPK-2 or MK2), a kinase involved in inflammatory responses, cell division and differentiation, apoptosis, and cell motility [6]. p38 is involved in apoptotic cell death and key molecules in the apoptotic onset (e.g., Bcl-2 superfamily members or p53) have been shown to be p38 substrates [99,100]. Furthermore, MK2 is activated after DNA damage [101,102] resulting in cell cycle arrest and ultimately cellular senescence. These characteristics of p38 and MK2 make them attractive targets for chemotherapy considering that apoptosis and DNA repair are the main mechanisms associated with cell survival during DNA damage [103], thus a possible role in cancer treatment is being explored [104].

Many cancer cells have abrogated G1 checkpoints due to lesions in tumor suppressor molecules such as p53 that regulate cellular senescence that can be a response to DNA damaging agents [35]. Nevertheless, such cancer cells often retain a G2 checkpoint, in particular a chromatin-quality checkpoint in late G2 involving ATR/p38/MK2 [101,102,105,106]. Genetic disruption of the p38/MK2 pathway can specifically sensitize p53-null mouse cells to DNA damaging agents [107,108]. It seems that p53-null cells with abrogated p38/MK2 pathways have lost both G1 and G2 DNA damage checkpoint function and enter mitosis in the presence of DNA damage, where they die by “mitotic catastrophe”. In contrast, p53 wild-type cells can still arrest in response to DNA damage as the p53-dependent G1 checkpoint is still active. These cells halt in G1 and do not enter into mitotic catastrophe. This suggests that small molecule targeting p38 or MK2 may result in sensitizing tumor cells to chemotherapeutic drugs.

Several p38 and MK2 inhibitors have been tested for their ability to increase the effectiveness of chemotherapeutic agents. For example, p38 activity was inhibited in gastric cancer cells (BGC823) using SB203580 that improved the sensitivity BGC823 cells to doxorubicin and induced cell death [109]. Furthermore, co-treatment with SB202190 and irinotecan improved the sensitivity of chemoresistant colorectal cancer cells to chemotherapy [110], and a p38α-selective MAPK inhibitor SCIO-469 reduced tumor growth in multiple myeloma xenograft tumors by enhancing the effect of bortezomib [6]. The MK2 pathway has been less studied compared to p38, however, some data suggest that the MK2 inhibitor MK2.III increases the sensitivity of pancreatic cancer cells to gemcitabine [111], and recent data show that MK2 knockdown reduces in vivo growth of multiple myeloma in mouse models with MK2 overexpression leading to bortezomib and doxorubicin chemoresistance by reducing apoptosis [112]. All these data suggest a role for p38 and MK2 in chemoresistance making them attractive targets for further research.

## 3. Anti-Apoptotic Mechanisms in Resistance to Chemotherapy

### 3.1. Apoptotic Cell-Signaling Pathways

**Figure 1:**
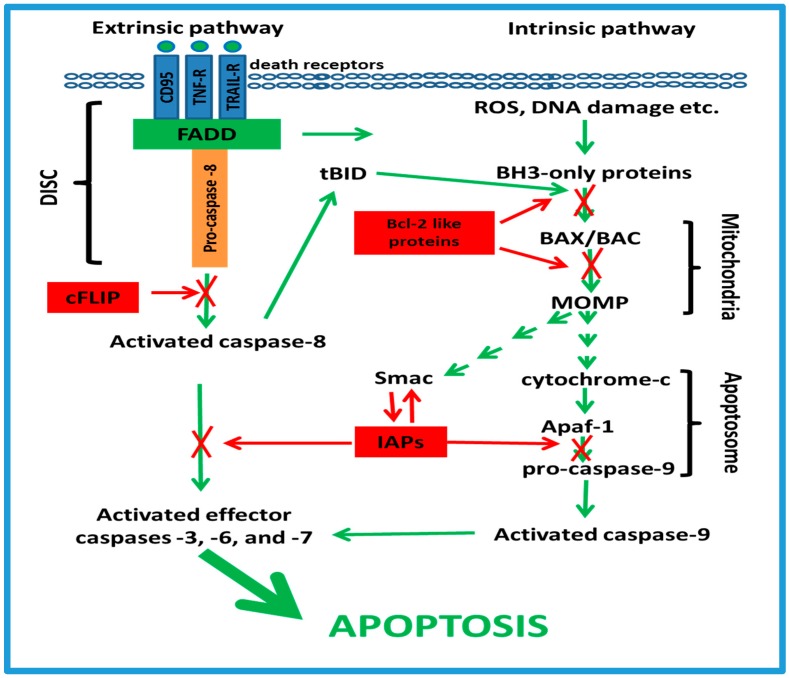
Anti-apoptotic mechanisms contributing to chemotherapy resistance in cancer cells. Extrinsic apoptotic pathways in tumors may be suppressed by downregulation of cell surface death receptors or/and overexpression of cFLIP; intrinsic (mitochondrial) apoptosis is blocked by up-regulation of Bcl-2-like proteins, and both pathways can be terminated by IAPs. FADD—FAS associated protein with death domain; DISC—the death-inducing signaling complex; cFLIP—the cellular FLICE (caspase 8)-like inhibitory protein; tBID—truncated Bid protein; MOMP—mitochondrial outer membrane permeabilization; Smac—second mitochondria-derived activator of caspases; Apaf 1—apoptotic protease activating factor 1; IAPs—inhibitor of apoptosis proteins. TRAIL. Note: green arrows—activation, red arrows—suppression, X—blocking.

There are two primary apoptotic cell-signaling pathways: extrinsic and intrinsic (Figure 1). The extrinsic pathway is triggered via interaction of cell surface death receptors, including CD95 (First apoptotis signal receptor/apoptosis antigen-1 [FAS-R/APO-1]), tumor necrosis factor-receptor (TNF-R) and TRAIL-receptors (TRAIL-Rs) with their death ligands, CD95L, TNF, or TRAIL [113]. Binding of death ligands leads to the formation of the death-inducing signaling complex (DISC) that involves the sequential recruitment of FAS associated protein with death domain (FADD) and pro-caspases to the receptor cytosolic domain, and subsequent activation of initiator caspases (e.g., caspase-8, caspase-10) [113]. Once activated, initiator caspases trigger downstream effector caspases (caspase-3, -6, and -7) that, in turn, cleave vital cellular proteins responsible for the characteristic biochemical and morphological hallmarks of apoptosis [114].

The second pathway leading to programmed cell death is the intrinsic mitochondria-dependent apoptotic pathway (Figure 1). The essential initiators of this pathway are the BH3-only proteins Bid, Bim, Puma, Noxa, Bad, Bmf, Hrk, and Bik. Upon activation by intracellular stimuli, such as excessive ROS, DNA damage, and the unfolded protein response, BH3-only proteins translocate to mitochondria and activate the pore-forming Bax/Bak proteins at the mitochondrial outer membrane [115]. This leads to membrane permeabilization and the release of second mitochondria-derived activator of caspase/Direct Inhibitor of Apoptosis-Binding protein with Low pI (Smac/DIABLO) and cytochrome-c from the inter membrane space into the cytoplasm. Bax/Bak is also activated by caspase-8 via the BH3-only protein tBID [116]. The release of cytochrome-c binds to the cytosolic proteins Apaf-1 and pro-caspase-9 to facilitate the formation of the apoptosome. The apoptosome activates initiator caspase-9 and, consequently, effector caspases, thus triggering apoptosis [115]. A second protein released from the intermembrane space, Smac/DIABLO, suppresses the inhibitor of apoptosis proteins (IAPs) that negatively regulate the activity of initiator and effector caspases [117].

However, in malignant cells, intrinsic or acquired up-regulation of anti-apoptotic mechanisms and/or down regulation of pro-apoptotic molecules allows cancer cells to escape apoptosis and develop resistance to chemotherapy [103]. This resistance is a complex phenomenon that involves the interactions of various molecules and signaling pathways. In this section we discuss several anti-apoptotic mechanisms contributing to chemotherapy resistance in cancer cells, and pharmacological approaches that may help to increase susceptibility of tumors to anti-cancer treatment.

One of the cytokines associated with apoptosis and expressed in various tissues is TRAIL, a protein related to the superfamily of tumor necrosis factors. Death receptors DR4 and DR5 in cancer cells have high-affinity binding of TRAIL and their receptor/ligand interactions induce extrinsic apoptotic cell-signaling pathway [118]. In comparison with CD95/CD95L and TNFα/TNF-R this ligand cannot provoke immunoinflammatory response as lethal septic shock like side effect. In addition, the CD95/CD95L and TNFα/TNF-R1 have been reported to activate the oncogenic NF-κB pathway, while TRAIL has no, or a weak, effect on NF-κB activation and, therefore, it is considered a prospective chemotherapeutic agent [119]. However, TRAIL therapy is limited by resistance in a large number of cancer cells due to intrinsic or acquired downregulation of TRAIL-Rs [120] and sensitization of TRAIL/TRIAL-Rs axis via different cellular mechanisms of great clinical interest.

For example, monesin, medicarpin, diallyl trisulfide, tunicamycin, and 5,7-dimethoxyflavone initiated up-regulation of death receptor 5 (DR5) in glioma, myeloid leukemia, human melanoma and hepatocellular carcinoma cells via the unfolded protein response [121,122,123,124,125]. Addition of proteasome inhibitor PS-341 (VELCADE, bortezomib) sensitized prostate cancer cells to TRAIL-induced apoptosis by increasing DR5 inhibiting protein degradation, and elevating *DR5* mRNA [126]. TRAIL-resistant prostate cancer cells, glioma and HeLa cells have also been sensitized by inhibition of heat shock proteins 90 and 27 with geldanamycin, and small molecules 17-AAG and LY303511 [127,128,129]. In addition, targeting p53 [130], autophagy [131], protein synthesis [132] and epigenetic modulation [133] by different agents have been reported to increase sensitivity of breast, myeloid, lung, brain, skin, cervical and colon cancer cells to TRAIL therapy.

### 3.2. The cFLIP Proteins

Besides FADD and the procaspase-8, the cellular FLICE (caspase 8)-like inhibitory proteins (cFLIP) can be recruited to the DISC (Figure 1). Three human cellular homologs of cFLIP have been identified: c-FLIP(L), c-FLIP(S) and c-FLIP(R), which are generated by differential splicing [134]. Unlike procaspase-8, cFLIP proteins lack a catalytic cysteine in their active center, but are capable of binding to FADD and/or caspase-8 or -10 in a ligand-dependent manner and preventing further activation of the caspase cascade [135,136] (Figure 1). Upregulation of cFLIP has been shown in various cancer cells and is implicated in chemoresistance in response to various anticancer drugs [137,138,139,140,141]. In turn, inhibition of c-FLIP can significantly improve susceptibility of malignant cells to chemotherapy.

It has been demonstrated that chemoresistant murine thymoma cell lines were sensitized to CD95-induced apoptosis by cycloheximide via downregulation of cFLIP(L) [136]. Similarly, the motif chemokine (CXC) chemokine receptor (CXCR2) antagonist Z10397767 attenuated interleukin-8 (IL-8) induced c-FLIP(S) up-regulation in prostate cancer cell lines thus enhancing sensitivity of these cells to TRAIL-chemotherapy [142], and thioridazine increased susceptibility of head and neck squamous cell carcinoma cells (AMC-HN4) to carboplatin through downregulation of c-FLIP and Mcl-1 expression [143]. Additionally, it has been demonstrated that degradation of c-FLIP by the histone deacetylase inhibitor LBH589 and a steroidal lactone from Physalis peruviana Withanolide E mediated sensitization of pancreatic cancer cells and renal carcinoma cells to TRAIL-induced apoptosis [144,145] (Table 1).

### 3.3. The Bcl-2-Like Proteins

The intrinsic pathway can be suppressed by overexpression of pro-survival Bcl-2-like proteins such as Bcl-2, Bcl-XL, Bcl-W, Mcl-1 and Bfl-1/A1 [146,147,148]. In turn, there is data indicating that suppression of Bcl-2-like proteins leads to the activation of apoptosis in chemoresistant cancer cells (Table 1), whereas their up-regulation in tumor cells is associated with poor prognosis and resistance to chemotherapy [146,147,148]. For example, inhibition of Bcl-2 and Bcl-XL by the small molecule compound ABT-737 significantly increased the number of apoptotic cells without affecting proliferation in human colorectal tissue cultured ex vivo [148]. Similarly, the small molecule Bcl-2-like protein inhibitor ABT-263 (navitoclax) exhibited single-agent antitumor activity in murine models of small cell lung cancer, leukemia, and lymphoma, and enhanced cytotoxicity when used with docetaxel or erlotinib in xenograft models [147]. Furthermore, the small molecule S63845 that specifically binds to Mcl-1 activates the Bax/Bak-dependent mitochondrial apoptotic pathway in myeloma, leukemia and lymphoma cells [149,150]. There is also data indicating that the small molecule ML214 (4-chloro-1-methyl-3-nitroquinolin-2-one) induces caspase activation in mouse embryonic fibroblasts (MEFs) overexpressing either A1-2A-BIM or A1 and alternate pro-apoptotic Bcl-2 protein tBID. In addition, this compound induces caspase activation and cytochrome c release in human melanoma cell line expressing A1-2A-BIM [151].

### 3.4. The IAP Family

Another large group of proteins implicated in cancer cell apoptotic resistance are the inhibitors of apoptosis proteins (IAP) family [152,153,154,155]. There are eight members of this family identified in humans: neuronal IAP/NAIP (BIRC1); cellular IAP1, also called cIAP1/HIAP2 (BIRC2); cellular IAP2, also called cIAP2/HIAP1 (BIRC3); X-linked IAP/XIAP/hILP (BIRC4); Survivin (BIRC5); BIR containing ubiquitin conjugating enzyme/BRUCE/Apollon (BIRC6); Melanoma IAP/ML-IAP/Livin (BIRC7); and IAP-like protein 2/hILP2/Ts-IAP (BIRC8). The IAPs regulate the activity of initiator and effector caspases [117], and suppression of IAPs can augment the apoptotic effect of chemotherapeutics for many types of cancer cells (Table 1).

Moon et al. have shown that the novel small-molecule ZAD5582 promoted TNF-α-induced apoptosis through targeting cIAP1 and XIAP in human pancreatic cancer cells [156]. AEG35156, a novel second-generation antisense oligonucleotide directed towards XIAP, increased sensitization of pancreatic carcinoma cells to TRAIL–mediated apoptosis as a single agent and was capable of inducing complete tumor regression when combined with taxanes in three human cancer xenograft models (prostate, colon, and lung) [157]. A Phase I clinical trial of AEG35165 demonstrated clinical evidence of its antitumor activity in patients with advanced refractory cancers [158]. Gyuraszova et al. have demonstrated that the small-molecule YM155, which is an inhibitor of BIRC5 (survivin), was capable of potentiating the cytotoxic effect of hypericin-mediated photodynamic therapy (HY-PDT) in two cell lines resistant to HY-PDT, HT-29 (colorectal adenocarcinoma) and A549 (lung adenocarcinoma) [159].

As mentioned, the Smac/DIABLO is a second protein released from the intermembrane space upon the outer membrane permeabilization, and it is an endogenous antagonist of XIAP, cIAP1, and cIAP2. In the last decade, small-molecule Smac mimetics have been developed to induce death receptor-mediated cell death activity. Several research groups have shown that Smac mimetics are capable of targeting multiple IAPs and increasing apoptosis in TRAIL-resistant breast cancer cell lines [160,161]. Combination of Smac mimetics with inhibitors of platelet-derived growth factor receptor (PDGFR), insulin-like growth factor 1 receptor (IGF1R) and epidermal growth factor receptor (EGFR) significantly increases apoptotic cell death compared to monotherapy in human glioblastoma cells [162], and combined therapy of Smac mimetics with inhibitors of FMS-like tyrosine kinase 3 (FLT3) and BCR-ABL resulted in similar effects against leukemia [163,164].

Thus, inhibition of both the extrinsic and intrinsic apoptotic pathways is implicated in chemoresistance in many cancer cells. This chemoresistance may be due to downregulation of cell surface death receptors and/or up-regulation of anti-apoptotic proteins such as Bcl-2 like, cFLIP, or IAPs, making the targeting of those molecules an attractive and important task in the development of the next generation cancer chemotherapies (Figure 1).

## 4. Transforming Growth Factors

Transforming growth factor (TGF) is a signaling pathway for the family of cytokines that includes the polypeptide growth factors TGF-α and TGF-β. Depending on the number of polypeptide chains, they act through different receptor mechanisms in vitro and in vivo [172]. TGF-α, a single chain polypeptide, stimulates epidermal growth factor by activating the epidermal growth factor receptor (EGFR), while the two chain polypeptide TGF-β is associated with proliferation, differentiation, renewal of stem-like cell populations and invasion [173]. Furthermore, TGF-β consists of both similarity to Mothers against decapentaplegic (SMAD)-dependent and independent pathways [174]. Although both growth factors are upregulated in many cancers, TGF-β and its isomers (TGF-β1, TGF-β2, TGF-β3) have been the most studied pathways in cancer treatment, possibly due to their dual action during cancer progression. It is well known that TGF-β acts as tumor suppressor in healthy cells or at early stages of cancer development by inducing growth arrest and apoptosis, however it promotes cancer progression by initiating tumor cell migration and stimulating epithelial to mesenchymal transition (EMT) at later phases of cancer [175,176]. Recent studies have indicated EMT’s association with TGF-β induced cancer/tumor progression, chemoresistance and metastasis [177,178]. The mechanistic action for TGF-β to drive drug resistance during chemotherapy appears to be its overactivation, promotion of EMT, regulation of DNA repair and initiation of cell cycle arrest and autophagy [177]. TGF-β downregulation has been linked to overcoming doxorubicin resistance in HCT116 colon cancer cells [178] and is shown to exert oxaliplatin resistance in colorectal cancer [177], high resistance of breast cancer cells to doxorubicin through EMT overexpression [179] as well as resistance of triple negative breast cancer cells to paclitaxel [180].

Several combinatorial strategies are being employed aimed at overcoming chemoresistance of cancer cells during chemotherapy involving TGF-β inhibition. A number of ALK5 inhibitors such as EW-7195, EW-7203 and IN-1130 have shown to effectively block TGF-β1-induced SMAD signaling, EMT and breast cancer metastasis in vivo [181,182,183], demonstrating the potential of these small molecules to prevent breast cancer metastasis to the lung. Another TGF-β type I receptor kinase inhibitor LY2157299 also blocked paclitaxel-induced IL-8 transcription and cancer stem-like cell expansion in vivo during triple negative breast cancer treatment using mouse xenograft models [180]. Furthermore, combined therapy with sorafenib (tyrosine kinase inhibitor) and galunisertib resulted in elevated apoptosis and low proliferation level in hepatocellular carcinoma slices [184] while TβRI inhibitor LY364947 and erlotinib treatment led to decreased motility rate of NSLCL cells [175].

## 5. Role of Cell Adhesion Molecules (CAMs) in Chemoresistance

A growing body of evidence indicates that cell-cell and cell-extracellular matrix (ECM) contacts can modulate drug resistance and the phenomenon is referred to as cell adhesion-mediated drug resistance (CAM-DR) [185,186]. Most mechanisms of CAM-DR are not fully understood, possibly due to multifactorial processes involving different molecular players such as cell adhesion molecules and ECM components. The literature indicates that multidrug-resistant phenotypes of cancer cells are associated with cell adhesion molecules that play a key role in cell adhesion processes [186]. An increasing number of studies demonstrate that different types of CAMs may be involved in the resistance to chemotherapy treatment [16,185,187,188,189,190]. This section discusses the literature focusing on CAMs as attractive therapeutic targets to overcome chemoresistance in cancer therapy.

The broad spectrum of CAMs is classified into different families, although most belong to four principal classes: Ig (immunoglobulin) superfamily (IgSF CAMs), integrins, cadherins and selectins. Some unclassified CAMs including CD 44 and epithelial cell adhesion molecule (EpCAM), are considered as separate to the above four classifications [191,192,193]. Over the past decade, a number of studies have demonstrated that many CAMs are overexpressed in chemotherapy-resistant cancer cells, whereas some CAMs, such as integrin α2β1, CD31 and E-cadherin under expressed in cancer cells [16,194]. The loss of E-cadherin expression is related to chemoresistance in response to paclitaxel and docetaxel in prostate cancer [195]. It was shown that EMT reduces E-cadherin expression via EMT inducers [196]. EMT leads to docetaxel resistance in prostate cancer and decreases E-cadherin expression associated with disease relapse [197].

Numerous studies have demonstrated that cell-adhesion mediated drug resistance (CAM-DR) is based on a number of mechanisms. For example, protecting cells from drug-induced cytotoxic damage in cancer cells with CAM-DR was achieved by enhancing the repair of DNA damage due to overexpression of histone methyltransferase multiple myeloma SET domain (MMSET) in multiple myeloma [198,199], or elevated expression of FANCF and RAD51C which are important DNA repair proteins [187,200,201,202,203,204]. Another mechanisms of CAM-DR is increasing resistance to detachment-induced cell death (anoikis resistance), and inhibition of FAS-mediated apoptosis [205,206,207,208].

Despite these findings, the mechanisms involved in cell-adhesion mediated drug resistance are not fully explored. Accumulating evidence points to a critical role of post-transcriptional processes and the epigenetic modifications that can induce drug resistance [199].

There are some attractive features of anti CAM-DR strategy. Thus, studies mentioned above have revealed that abnormal expression of cell adhesion molecules is frequently associated with aggressive tumor growth, metastasis and resistance to chemotherapy [209]. Moreover, modification and/or disruption of E-cadherin was detected at early stages of tumor development [210,211,212] and also CD44 is involved in an early step necessary for metastasis [213]. It can be assumed that regulation of CAMs can prevent the development of acquired mechanisms of drug resistance.

These findings indicate that cell adhesion molecules may serve as a target for personalized cancer therapy due to their frequent expression in different types of neoplasms, specific distribution in normal cells and dysregulated before the invasion [186]. Moreover, new molecular insight such as whole genome sequencing reveals that frequent somatic mutations in gastric cancers [214], lung squamous cell carcinoma [215] and leukemia [216] were in cell adhesion genes. Therefore, targeting CAMs introduces special interest for scientists and pharmacological companies as well as for the development of anti-CAM-DR strategy to sensitize cancer cells to therapeutic drugs [186,217].

Most research targeting CAM-DR has focused largely on integrins [202,218,219,220,221,222,223,224,225,226,227,228,229,230,231,232,233,234,235,236,237,238,239,240,241] (see Table 2). Considerable efforts have been directed at examining the inhibitory action of integrin agonists, such as antibodies, peptides and small molecules [218,229,230]. Different preclinical in vitro and in vivo studies showed that targeting α4 integrin by antibodies sensitizes multiple myeloma to chemotherapy using melphalan or bortezomib [223,237] (Table 2), and α4 integrin small molecule inhibitor TBC3486 increases acute lymphoblastic leukemia sensitivity to vincristine treatment [221,222]. There is only one known Phase I/II clinical trial (NCT00675428) in patients with refractory multiple myeloma treated by natalizumab, a recombinant humanized IgG4 monoclonal antibody, which binds integrin α4. However, this clinical trial was terminated due to low enrollment.

The synthetic Arg-Gly-Asp-motif peptide integrin α5α3 inhibitor EMD-121974 (cilengitide), was a very attractive drug for anti-CAM-DR strategy as it was shown to demonstrate a positive outcome in preclinical studies [236]. Cilengitide was tested in several clinical trials for different types of tumors, including glioma, NSCLC and squamous cell carcinoma [231,232,233,234,235]. Despite positive results from preclinical studies and second phase clinical trials, the addition of cilengitide to temozolomide chemo and radiotherapy did not improve patient’s overall survival with newly diagnosed glioblastoma in an EORTC Phase III randomized, controlled, multicenter clinical trial [232]. ExCentric, a multicentre open-label Phase II trial, showed that cilengitide did not enhance survival of MGMT-promoter unmethylated glioblastoma when used in combination with procarbazine and metronomic temozolomide compared with historical data [235]. Results of the randomized Phase I/II ADVANTAGE trial (Phase II part) demonstrated that cilengitide with cetuximab and platinum-based chemotherapy in recurrent/metastatic squamous cell carcinoma of the head and neck did not result in any positive outcome [234]. Cilengitide combined with cetuximab and platinum-based chemotherapy was tested in an open-label randomized controlled Phase II study (CERTO) as first-line treatment for patients with advanced non-small-cell lung cancer (NSCLC) [233]. The study showed that patients with advanced NSCLC had improved progression-free survival rate compared with control.

It was shown that the 3-hydroxy-3-methylglutaryl-coenzyme (HMG-CoA) reductase inhibitor simvastatin can selectively inhibit integrins, shows antimyeloma activity and up-regulates HMG-CoA reductase in chemotherapy-resistant cancer cells [238,239,240]. Schmidmaier and co-authors showed that simvastatin at very low concentrations overcomes CAM-DR in multiple myeloma by geranylgeranylation of Rho proteins and activation of Rho kinase [228]. In addition, simvastatin diminishes tumor cell adhesion to human peritoneal mesothelial cells by reduced expression of VCAM-1 and β1 integrin [241]. Simvastatin as an inhibitor of CAM-DR in patients with refractory multiple myeloma was tested in Phase II clinical trials by Ludwig-Maximilian University of Munich. This clinical trial demonstrated suppression of drug resistance by inhibition of HMG-CoA-reductase [17].

Although most studies in this area have focused on integrins, other CAMs have potential for anti-CAM-DR strategy. Thus, recent data showed that down-regulation of endothelial adhesion receptor CD31/PECAM-1 (platelet endothelial cell adhesion molecule-1) was associated with resistance against oxidative stress and DNA damage in angiosarcoma cells due to YAP (yes-associated protein) signaling, and inhibition of YAP by pazopanib re-sensitized cancer cells to doxorubicin [242]. Pazopanib may find use as a CAM-DR inhibitor as it inhibits VEGF-induced up-regulation of adhesion molecules on tumor cells [243]. Pazopanib maintenance therapy provided a statistically significant and clinically meaningful progression-free survival (PFS) benefit in patients with advanced epithelial ovarian, fallopian tube, or primary peritoneal cancers in Phase III trials [244].

The intercellular adhesion molecule E-cadherin is considered as a key player in the process of acquiring chemoresistance [188,195,210,212,245]. Down regulation of E-cadherin has been noted in many human cancers [210], and is associated with chemoresistance in prostate cancer cells (PCa) [195]. Overexpression of E-cadherin in chemoresistant PCa cells inhibited cell migration and invasion and increased their sensitivity to paclitaxel [195]. In addition, docetaxel treatment can lead to E-cadherin down regulation leading to poor prognosis in prostate cancer [245]. It is suggested that docetaxel treatment leads to a clonal selection of highly invasive prostate cancer cells thus leading to chemosresistance [245]. This chemoresistance may be due, at least in part, to the acquisition of a mesenchymal and stem cell-like phenotype. These data suggest the development of drugs allowing E-cadherin re-expression may have novel therapeutic possibilities.

Recently, it was shown that growth promoting Notch signaling has a role in E-cadherin associated cancer chemoresistance [195], and Notch pathway up-regulation has been observed in PCa clinical samples [246]. An inverse correlation between E-cadherin and Notch-1 expression was seen in chemoresistant PCa cells, and treatment of these cells with a γ-secretase inhibitor (GSI) restored chemosensitvity to paclitaxel [195]. The γ-secretase activity is required for the final cleavage step of the precursor form of Notch to activate Notch signaling [247]. Inhibiting Notch signaling may also be of benefit in other cancer types, as the GSI MK-0752 combined with docetaxel improved the health of patients with advanced breast cancer in Phase II clinical trials [248]. These initial results of preclinical studies suggest inhibitors of Notch signaling as potential anti CAM-DR drugs and further studies are warranted.

There is increasing evidence that selectins play an important role in the progression of different types of cancer, in particular the interaction of tumor cells with the endothelium that is needed for extravasation and the formation of new metastatic lesions [249,250] Selectins are molecules expressed on the cell surface of endothelial cells that have been shown to promote the first interaction between an extravasating cell and the blood-vessel wall [251], and have been implicated in CAM-DR [252,253,254,255]. As metastasis can be dramatically reduced in mice deficient for P- and L-selectins, this suggests selectins as possible drug targets for chemosensitization [256], and it has been reported that small molecule glycomimetic selectin antagonists have the ability to sensitize cancer cells [252,253,254,255]. For example, the small molecule pan-selectin inhibitor GMI-1070 enhances the sensitization of multiple myeloma cells to bortezomib, both in vitro and in vivo, by targeting P-selectins [252]. Similar effects were seen when P-selectins were blocked using monoclonal antibodies [254], and the addition of E-selectin inhibitor GMI-1271 to induction chemotherapy in elderly patients with untreated acute myeloid leukemia demonstrated a high remission rate and low mortality [255,257]. In addition, pulmonary metastasis of melanoma cells was shown to be reduced in vivo using the P-selectin ligand antagonist holothurian glycosaminoglycan [253]. These recent studies provide support that targeting the selectins may be a good strategy for chemosensitization.

CD44, a transmembrane receptor for hyaluronan, is a functional component of cell adhesion-mediated drug resistance, as its blockade was shown to sensitize drug resistant multiple myeloma to lenalidomide [258,259]. In addition, Zheng et al. recently reported that small molecule aurora kinase inhibitors attenuated breast tumor-initiating cells and overcame epirubicin resistance by CD44 inhibition [260]. These data suggest that CD44 should be evaluated as a putative biomarker of sensitivity to various chemotherapeutic drugs.

These data strongly support that translation of CAM-DR signaling pathway regulators to clinic in combination with standard therapy can be considered as a rational strategy to overcome resistance. However, in comparison with the significant advances in other strategies for overcoming chemoresistance, progress made with respect to anti CAM-DR therapy has been relatively slow. A major therapy failure was a Phase III clinical trial using cilengitide in newly diagnosed glioblastoma [232]. Despite the challenges in this area and gaps in our knowledge, our understanding is that the development of effective strategies for overcoming chemoresistance will require a better understanding of cell adhesion drug resistance and its interaction with other types of chemoresistance. Table 2 summarizes CAM signaling pathways involved in chemoresistance.

## 6. Conclusions

Cancer cells can escape the toxic effects of chemotherapy through a variety of mechanisms such as cell cycle, apoptosis and cell adhesion. Studies reveal that the mechanisms by which cancer cells mediate chemoresistance can involve various oncogenic factors and several different signaling pathways. However, these mechanisms can be targeted using specific inhibitors that may improve the sensitivity of cancer cells to chemotherapeutic agents when used in combination. Studies highlighted in this review provide evidence that specific inhibitors of components in key signaling pathways involved in chemoresistance may indeed improve overall cancer therapy. With a more personalized medical approach to cancer envisioned for the future, an in-depth knowledge of chemoresistance mechanisms in specific cancer types together with proper diagnosis is required; this will hopefully lead to a more targeted and informed cancer treatment, and prove a useful strategy to overcome drug treatment failures that ultimately lead to recurrence and death.

## Figures and Tables

**Table 1:** Cellular targets for the inhibition of anti-apoptotic mechanisms in cancer.

Target Proteins		Anti-Apoptotic Mechanisms	Inhibitors	Type of Tumor
The cellular FLICE-like inhibitory protein (cFLIP)		Competitive interference with caspase-8 recruitment to DISC	Cycloheximide [136]	murine thymoma EL-4 cells
			miRNA-708 [165]	renal cancer cells
			CXCR2 antagonist Z10397767 [142]	prostate cancer cells
			Thioridazine [143]	human head and neck cancer cells
			Histone deacetylase inhibitor LBH589 [144]	pancreatic cancer cells
			Withanolide E and analogues [145]	renal carcinoma
**Bcl-2-like proteins**	Bcl-2 Bcl-XL Bcl-W	Inhibition of pore-forming Bax/Bak in mitochondria	Small-molecule ABT-737 [148]	human colorectal cancer
			Small-molecule ABT-263 (navitoclax) [147]	small cell lung cancer leukemia lymphoma hematologic malignances
	Mcl-1	Antagonizes Bax and Bak activation	Small-molecule S63845 [149]	myeloma leukaemia lymphoma
	Bfl-1/A1	Binds to BH3-only proteins [166]	4-chloro-1-methyl-3-nitroquinolin-2-one [151]	MEF and melanoma cell lines primed with various A1 constructs
**IAPs**	BIRC4 (X-linked IAP/XIAP/hILP) BIRC2 (cellular IAP1/cIAP1/HIAP2) BIRC3 (cellular IAP2/cIAP2/HIAP1)	Prevent downstream proteolytic processing of pro-caspase-3, -6 and -7 [117,167]	AZD5582 [156]	pancreatic cancer
			Oligonucleotide AEG-35156 [157]	Panc-1 pancreatic carcinoma cells, xenograft models of prostate, colon, and lung cancer, lymphoma, melanoma, breast cancer,
			Smac mimetics [160,161,162,163,164,168].	breast cancer multiple myeloma human glioblastoma non-small cell lung cancer leukemia
			Small-molecule AEG40730 [169]	HCT116 Cell Line human colon carcinoma
	BIRC5 (Survivin)	Binds to pro-caspase-9, preventing its recruitment to Apaf1 [170] Inhibits SMAC [171]	Small-molecule YM155 [159]	colorectal and lung adenocarcinoma
**Serine/threonine protein kinases**	WEE1	Dysregulates CDK1 and CDK2	MK1775 (AZD 1775)	ovarian cancer advanced gastric adenocarcinoma metastatic solid tumors [47]

**Table 2:** Regulation of drug resistance related to cell adhesion molecules.

Type of CAM	Type of Tumor	Chemotherapy Drugs	Signaling Pathway	Preclinical Anti CAM-DR Treatment	Clinical
Integrin α4	acute lymphoblastic leukemia	vincristine	Direct inhibition	Integrin α4 inhibitor small molecule TBC3486 [221,222]	-
	multiple myeloma	melphalan	Direct inhibition	Anti-integrin α4 antibody [223]	-
Integrin α4	multiple myeloma	bortezomib	Direct inhibition	inhibitor Natalizumab, a recombinant humanized IgG4 monoclonal antibody, which binds integrin α4 [237]	Natalizumab—Phase I/II (NCT00675428)—multiple myeloma (terminated)
Integrin α4	Glioma cells	temozolomide	Direct inhibition	EMD-121974 (Cilengitide), a synthetic Arg-Gly-Asp-motif peptide-α4 integrin inhibitor [224]	Cilengitide with temozolomide—Phase III (NCT00689221)—newly diagnosed glioblastoma [232]
					Cilengitide combined with cetuximab and platinum-based chemotherapy (NCT00842712)—Phase II-non-small-cell lung cancer [233] Cilengitide with Cisplatin, 5-fluorouracil, and cetuximab (NCT00705016)—Phase I/II—squamous cell carcinoma of the head and neck [234]
					Cilengitide with metronomic temozolomide, procarbazine, and standard radiotherapy (NCT01124240)—Phase II [235]
Integrin α5β1	squamous cell carcinoma	cisplatin	Direct inhibition	Anti-α5β1 Integrin Neutral Antibody [225]	-
VLA-4 (integrin α4β1) and VLA-5 (integrin α5β1)	myeloma	doxorubicin and melphalan	Direct inhibition	Anti-VLA-4 VLA-5 antibody [226]	-
VLA-4 (integrin α4β1) and LFA-1 (integrin αLβ2)	multiple myeloma	melphalan, treosulfan, doxorubicin, dexamethasone, and bortezomib	HMG-CoA/GG-PP/Rho/Rho-kinase	Anti LFA-1 and VLA-4 antibodies. Geranylgeranyl transferase (GGTase) inhibitor GGTI-298 and Rho kinase specific inhibitors Y-27632. The HMG-CoA reductase inhibitor simvastatin [238,239,240].	Simvastatin with bortezomib, bendamustin dexamethasone—Phase II (NCT00399867) [227,228]—in Patients with Refractory Multiple Myeloma [17]
CD31/PECAM-1	Angiosarcoma	Doxorubicin	YAP	YAP inhibitors (Pazopanib) [242]	Pazopanib—Phase III NCT00866697- Ovarian, Fallopian Tube or Primary Peritoneal Adenocarcinoma [244]
E-cadherin	Prostate cancer	Paclitaxel	Notch	The γ-secretase inhibitor (GSI, a Notch inhibitor) [195]	The γ-secretase inhibitor MK-0752—Phase II-NCT00645333 Breast cancer
PSGL-1/P-selectin	In macrophage for macrophage-mediated myeloma drug resistance	Bortezomib	Direct inhibition	The pan-selectin inhibitor GMI-1070 [252]	-
P-selectin	Melanoma	-	Direct inhibition	P-selectin inhibitor- Holothurian glycosaminoglycan [253]	-
	Multiple myeloma	Bortezomib	Direct inhibition	Humanized Monoclonal Antibodies [254]	-
E-selectin	Multiple myeloma	Bortezomib	Direct inhibition	E-selectin inhibitor GMI-1271 [255]	E-selectin inhibitor GMI-1271-with mitoxantrone, etoposide and cytarabine Phase I/II NCT02306291-acute myeloid leukemia [257]
CD44	Breast cancer	epirubicin	Aurora kinase	Aurora kinase inhibitor AKI603 [260]	-
